# Long-term fate of bicuspid neoaortic valve after arterial switch operation

**DOI:** 10.1093/icvts/ivac059

**Published:** 2022-03-31

**Authors:** Yaroslav Ivanov, Tyson A Fricke, Edward Buratto, Igor E Konstantinov

**Affiliations:** 1 Department of Cardiac Surgery, Royal Children’s Hospital, Melbourne, VIC, Australia; 2 Department of Paediatrics, University of Melbourne, Melbourne, VIC, Australia; 3 Heart Research Group, Murdoch Children’s Research Institute, Melbourne, VIC, Australia; 4 Melbourne Centre for Cardiovascular Genomics and Regenerative Medicine, Melbourne, VIC, Australia

**Keywords:** Arterial switch operation, Bicuspid aortic valve

The incidence of the bicuspid pulmonary valve in the patients with the transposition of great arteries is reported between 1.2% and 7% [1, 2]. After the arterial switch operation, the pulmonary valve becomes neoaortic valve and is exposed to systemic pressure. Jeon *et al.*[3] reported long-term outcomes of the bicuspid neoaortic valve after arterial switch operation with emphasis on neoaortic root dimension and valve function. The incidence of the bicuspid pulmonary valve in their series was 5.1% (18/352). After propensity score matching, 2 groups were compared: bicuspid group (*n* = 15) and tricuspid group (*n* = 60) [3]. The long-term outcome showed that patients in the bicuspid group were more prone to significant aortic insufficiency compared with the tricuspid group with unchanged neoaortic root geometry in both groups. It appears that patients with bicuspid neoaortic valve are more prone to develop insufficiency. Although the exact cause of this observation is yet unclear, this could be related to either leaflet abnormality or root dilatation or a combination of the two.

Overall, we have recently demonstrated that neoaortic valve may become a problem in the long term after the arterial switch operation [4]. Of the 95 patients with >25 years of follow-up after the arterial switch operation, 15% had either at least moderate neoaortic valve insufficiency or had undergone neoaortic root surgery [4].

**Figure 1:**
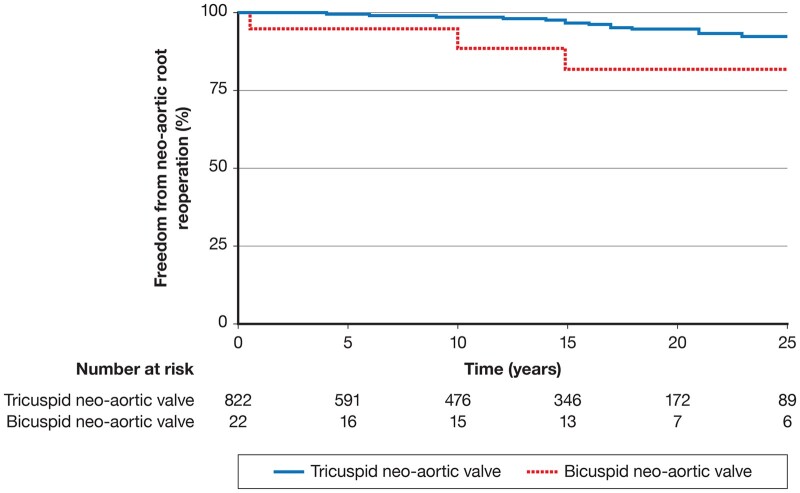
Freedom from neoaortic root reoperation in after arterial switch operation in patients with bicuspid neoaortic valves as compared to normal tricuspid neoaortic valve.

Similarly, the series from Melbourne include 844 patients with the transposition of great arteries operated from 1983 to 2015 [4]. The incidence of the bicuspid pulmonary valve was 2.6% (22/844). Reoperation on the neoartic root due to neoaortic valve insufficiency was required in 18% (4/22) of patients with bicuspid neoaortic valve compared to 3.6% (30/822) patients with tricuspid neoaortic valve (*P* = 0.01). Freedom from neoaortic root reoperation at 20 years was 82% for bicuspid neoaortic valve and 94% for tricuspid neoaortic valve (Fig. 1).

Thus, the data from Seoul and Melbourne suggest that morbidity associated with bicuspid pulmonary valve in the systemic circulation is not negligible. Therefore, the patients after arterial switch operation who have bicuspid neoaortic valve should have close follow-up due to higher than expected rate of neoaortic valve deterioration.
